# CD8^+^ T cell–derived CD40L mediates noncanonical cytotoxicity in CD40-expressing cancer cells

**DOI:** 10.1126/sciadv.adr9331

**Published:** 2025-05-21

**Authors:** Phillip Schiele, Alberto Sada Japp, Regina Stark, Joanna J. Sattelberg, Christos Nikolaou, Gereon Kornhuber, Parya Abbasi, Nina Ding, Stanislav Rosnev, Stefan Meinke, Kerstin Mühle, Lucie Loyal, Julian Braun, Manuela Dingeldey, Sibel Durlanik, Nadine Matzmohr, Donata Ponikwicka-Tyszko, Slawomir Wolczynski, Nafis A. Rahman, Ichiro Taniuchi, Stephan Schlickeiser, Claudia Giesecke-Thiel, Thomas Blankenstein, Il-Kang Na, Andreas Thiel, Marco Frentsch

**Affiliations:** ^1^ Therapy-Induced Remodeling in Immuno-Oncology, BIH Center for Regenerative Therapies (BCRT), Berlin Institute of Health at Charité–Universitätsmedizin Berlin, 13353 Berlin, Germany.; ^2^ Regenerative Immunology and Aging, BIH Center for Regenerative Therapies (BCRT), Berlin Institute of Health at Charité–Universitätsmedizin Berlin, 13353 Berlin, Germany.; ^3^ Captain T Cell GmbH, 12529 Berlin, Germany.; ^4^ Tissue Immunology, BIH Center for Regenerative Therapies, Charité–Universitätsmedizin Berlin, 13353 Berlin, Germany.; ^5^ Max-Delbrück-Center for Molecular Medicine and Institute for Immunology, Charité–Universitätsmedizin Berlin, 13125 Berlin, Germany.; ^6^ Si-M/“Der Simulierte Mensch,” Technische Universität Berlin and Charité - Universitätsmedizin Berlin, 13353 Berlin, Germany.; ^7^ Institute of Biomedicine, University of Turku, 20520 Turku, Finland.; ^8^ Department of Biology and Pathology of Human Reproduction, Institute of Animal Reproduction and Food Research, Polish Academy of Sciences, 10-748 Olsztyn, Poland.; ^9^ Department of Reproduction and Gynecological Endocrinology, Medical University of Bialystok, 15-276 Bialystok, Poland.; ^10^ RIKEN Research Center for Allergy and Immunology, Yokohama 230-0045, Japan.; ^11^ Institute for Medical Immunology, Charité-Universitätsmedizin Berlin, Corporate members of Freie Universität Berlin and Humboldt-Universität zu Berlin, Berlin, Germany.; ^12^ Max Planck Institute for Molecular Genetics, 14195 Berlin, Germany.; ^13^ Department of Hematology, Oncology and Tumor Immunology, and ECRC Experimental and Clinical Research Center, both Charité-Universitätsmedizin Berlin, Corporate members of Freie Universität Berlin and Humboldt-Universität zu Berlin, Berlin, Germany.; ^14^ German Cancer Consortium (DKTK), Berlin, Germany.; ^15^ ECRC Experimental and Clinical Research Center, Corporate Member of Freie Universität Berlin and Humboldt Universität zu Berlin, Charité Universitätsmedizin Berlin, Berlin, Germany.

## Abstract

T cells and their effector functions, in particular the canonical cytotoxicity of CD8^+^ T cells involving perforin, granzymes, Fas ligand (FasL), and tumor necrosis factor related apoptosis inducing ligand (TRAIL), are crucial for tumor immunity. Here, we reveal a previously unidentified mechanism by which CD40L-expressing CD8^+^ T cells induce cytotoxicity in cancer cells. In murine models, up to 50% of tumor-specific CD8^+^ T cells expressed CD40L, and conditional CD40L ablation in CD8^+^ T cells alone led to tumor formation. Mechanistically, CD40L^+^CD8^+^ T cells can induce cell death in CD40-expressing cancer cells by triggering caspase-8 activation. We demonstrate that a gene signature for resistance to CD40 signaling–induced cell death strongly correlates with worse survival in different human cancer cohorts. Our results introduce CD40L as a rather counterintuitive, noncanonical cytotoxic factor that complements the capabilities of CD8^+^ T cells to combat cancers and has the potential to enhance the efficacy of immunotherapies.

## Teaser

CD40L^+^CD8^+^ T cells reveal an unconventional way to kill cancer cells, offering the potential to boost immunotherapy effectiveness.

## INTRODUCTION

Canonical antitumor cytotoxicity by CD8^+^ T cells is mainly facilitated through the release of cytotoxic granules loaded with perforin and granzymes, whereas members of the tumor necrosis factor superfamily (TNFSF) such as Fas ligand (FasL), tumor necrosis factor–α (TNFα), and tumor necrosis factor related apoptosis inducing ligand (TRAIL) mediate death signals via extracellular receptor-ligand interactions, depending on the intracellular state of a target cell (*1*). We have previously demonstrated that a subset of activated CD8^+^ T cells expresses another TNFSF ligand, i.e., CD40L, although the precise biological function of this expression within CD8^+^ T cells remained unclear (*2*, *3*). In general, the expression of CD40L has been associated with the critical helper function of activated CD4^+^ T cells, facilitating essential CD40 signaling in dendritic cells, macrophages, and B cells crucial for their activation and differentiation (*4*–*6*). In line with this understanding, tumor antigen–independent CD40 agonists, such as anti-CD40 antibodies, have been explored in therapeutic strategies to boost tumor immunity by, e.g., overcoming the tumor-induced immunosuppression (*7*–*9*).

Beyond antigen-presenting cells, CD40 is found on various human cancer cell types, including carcinomas, melanomas, and B cell lymphomas (*10*–*14*). Unlike its supportive role in immune activation, CD40 signaling can trigger cell death in cancer cells, likely because of changes in cellular redox levels that occur with the malignant transformation of these cells (*15*, *16*). Previous studies showed that unlike anti-CD40 antibodies or soluble CD40L, the natural trimerized form of CD40L, e.g., expressed on the membranes of antigen-activated T cells, mediates cytotoxicity in cancer cells to a high degree (*17*–*21*). Given that only CD8^+^ T cells are capable of recognizing antigens presented on major histocompatibility complex class I (MHC-I), which are present on all somatic cells, CD40L expression by CD8^+^ T cells could potentiate anticancer immune responses against CD40-expressing cancer cells. However, the noncanonical cytotoxicity potential mediated by CD40L from CD8^+^ T cells has yet to be investigated.

Here, we demonstrate that CD40L expressed by CD8^+^ T cells can act as an essential component for proficient tumor immunity. CD40L expressed by activated CD8^+^ T cells interacts with CD40 on cancer cells and triggers cell death through caspase activation. Further, we could identify a gene signature in renal cell carcinoma (RCC) that predicts resistance to CD40-mediated cytotoxicity, which was associated with a much lower survival rate in patients of different cohorts. Our results propose CD40L on tumor-reactive CD8^+^ T cells as a critical factor to directly induce cancer cell death.

## RESULTS

### CD40L^+^CD8^+^ T cells are present in antitumor responses

**Fig. 1.**
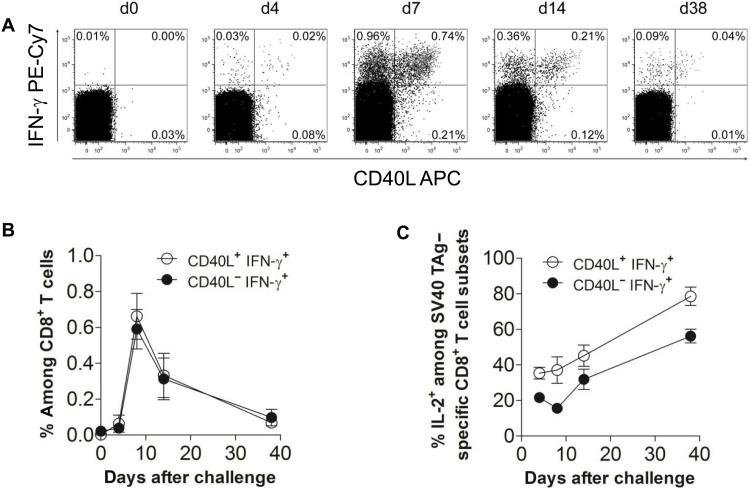
CD40L expression on SV40 TAg–specific CD8^+^ T cells during a protective immune response. (**A**) Splenocytes of WT mice challenged with 16.113 TAg^+^ cancer cells were stimulated with peptide IV at indicated time points. d, day. The dot plots show the intracellular IFN-γ and CD40L staining of CD3^+^CD8^+^CD4^−^-gated lymphocytes from one representative mouse (*n* = 4 mice). (**B**) The diagram summarizes the frequencies of CD40L^+^IFN-γ^+^ and CD40L^−^IFN-γ^+^CD8^+^ T cells and (**C**) the frequency of IL-2–producing cells among CD40L^+^ and CD40L^−^ tumor-specific CD8^+^ T cells at the different time points (both means ± SD).

To investigate the role of CD40L^+^CD8^+^ T cells in tumor control, we first assessed the capacity of tumor-reactive CD8^+^ T cells to express CD40L in an ectopic tumor model with SV40 T antigen (TAg)–expressing immunogenic cancer cells isolated from LoxPTAg mice (*22*, *23*). In TAg^+^ tumor models, CD8^+^ T cells are critical in the antitumor response, demonstrated by the successful rejection of 9.27 cancer cells in both wild-type (WT) and CD4-deficient (CD4^−/−^) mice but not in CD8^−/−^ or RAG1^−/−^ mice (fig. S1). For the analysis of TAg-reactive CD8^+^ T cell–derived CD40L, SV40 TAg–expressing cancer cells were injected into WT mice (*22*, *23*). Almost 50% of activated TAg-specific IFN-γ^+^CD8^+^ T cells expressed CD40L at all analyzed time points (Fig. 1, A and B). TAg-specific CD40L^+^IFN-γ^+^CD8^+^ T cells coexpressed more interleukin-2 (IL-2) as compared to TAg-specific CD40L^−^IFN-γ^+^CD8^+^ T cells (Fig. 1C) and remained present after rejection of cancer cells at day 38. This suggests that CD40L^+^CD8^+^ T cells constitute a polyfunctional, persistent cellular component of SV40 TAg–specific cellular tumor immunity.

### CD40L expressed by CD8^+^ T cells is pivotal for rejection of TAg-expressing cancer cells

**Fig. 2.**
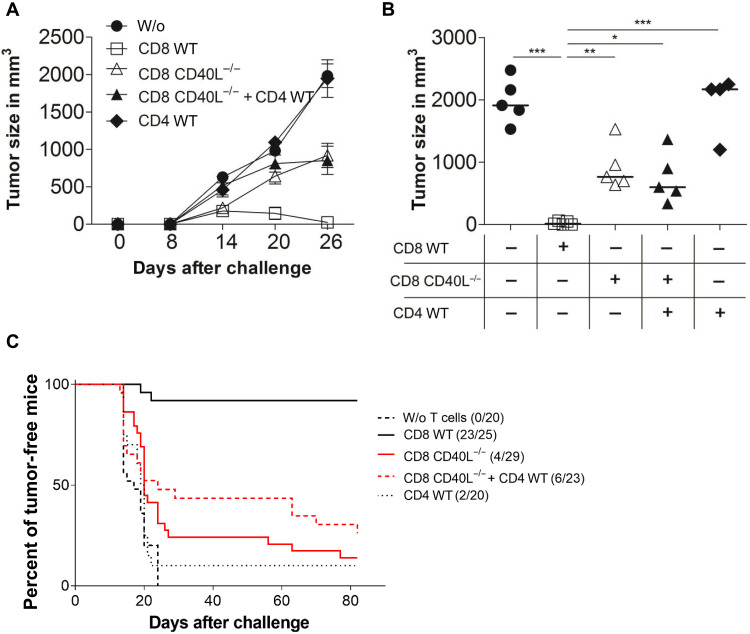
Prevention of tumor outgrowth is dependent on CD40L expression on CD8^+^ T cells. (**A**) RAG1^−/−^ mice were injected subcutaneously with 1 × 10^6^ 9.27 TAg^+^ cancer cells and treated in parallel with intravenously injected CD8^+^ T cells from WT or CD40L^−/−^ mice and/or with WT CD4^+^ T cells. The means ± SD of the tumor size of four or five mice per group are shown from one representative of five experiments. (**B**) The tumor sizes of individual mice in different groups are shown at day 26. (**C**) Summary of the tumor formation data obtained from five independent experiments. Tumor formation was defined as an established tumor when its volume reached >500 mm^3^. Statistical analysis: Analysis of variance (ANOVA) with Bonferroni multiple comparisons posttest: *P < 0.05, **P < 0.01, and ***P < 0.001.

To examine the tumoricidal function of CD8^+^ T cells, we transferred freshly isolated CD8^+^ T cells from WT or CD40L-deficient (CD40L^−/−^) mice into RAG1^−/−^ mice, challenged these mice with TAg^+^ cancer cells, and measured tumor growth. While the transferred WT CD8^+^ T cells rejected cancer cells in 23 of 25 mice, CD40L^−/−^CD8^+^ T cells failed to do so (4 of 29 mice; Fig. 2, A to C). The impaired antitumor response of CD40L^−/−^CD8^+^ T cells was not rescued by cotransferring WT CD4^+^ T cells, although they are capable to express CD40L upon activation. This demonstrates that the expression of CD40L by CD8^+^ T cells is essential in the rejection of TAg^+^ cancer cells. Besides affecting their capacity to control the cancer cells, CD40L deficiency in CD8^+^ T cells had otherwise no effect on their priming or functionality. Peripheral blood T cell count (fig. S2), in vivo cytotoxicity against peptide-loaded splenocytes (fig. S3), and the cytokine secretion profile of peripheral TAg-specific CD8^+^ T cells (fig. S4) were largely identical in RAG1^−/−^ mice reconstituted with either WT or CD40L^−/−^CD8^+^ T cells.

**Fig. 3.**
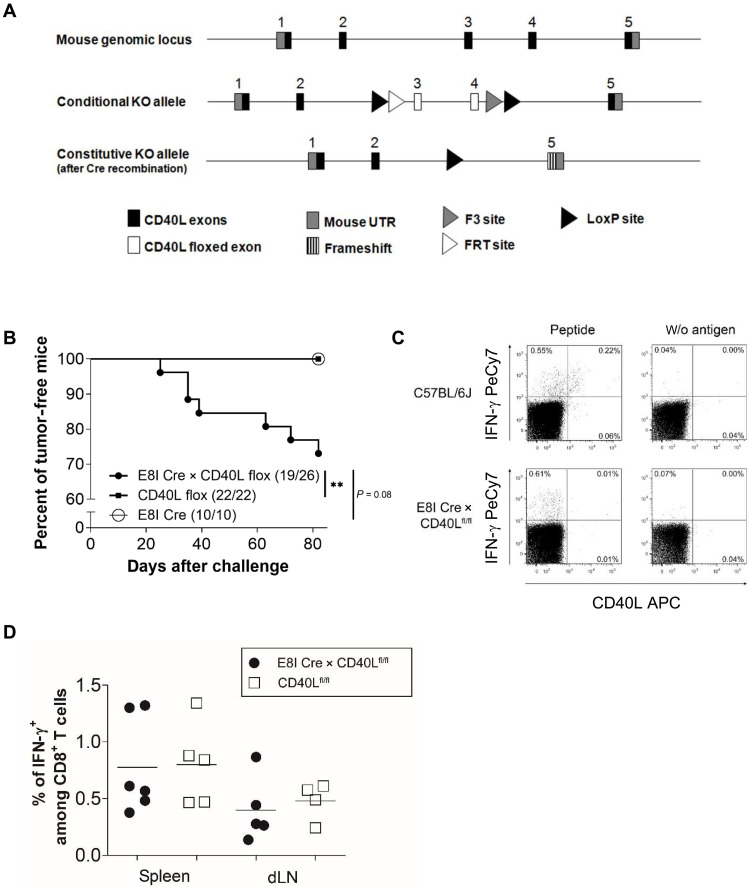
Impaired tumor rejection in nonlymphopenic CD8^+^ T cell–specific CD40L KO mice. (**A**) Strategy for the generation of CD40L^fl/fl^ mice. UTR, untranslated region; FRT, flippase recognition target. (**B**) E8I-Cre × CD40L^fl/fl^, E8I-Cre, and CD40L ^fl/fl^ mice as WT control were injected subcutaneously with 1 × 10^6^ 9.27 TAg^+^ cancer cells. Summary of the tumor formation data obtained from three individual experiments, each with 5 to 10 mice per group. Tumor was defined as established when its volume reached >500 mm^3^. (**C** and **D**) WT and E8I-Cre × CD40L^fl/fl^ mice were subcutaneously injected with 1 × 10^6^ 9.27 TAg^+^ cancer cells, and 7 days later, splenocytes and cells from the draining lymph nodes (dLNs) were isolated and stimulated with peptide IV. (C) The dot plots show the intracellular IFN-γ and CD40L staining of CD3^+^CD8^+^CD4^−^-gated splenocytes from one representative mouse of five mice. (D) The diagram summarizes the frequencies of TAg-specific IFN-γ^+^CD8^+^ T cells measured among splenocytes and lymph node cells. Statistical analysis: Log-rank test: **P < 0.01.

To rule out that lymphopenia in RAG1^−/−^ drives the observed CD40L-dependent cancer cell rejection by CD8^+^ T cells, we investigated the rejection of TAg^+^ cancer cells by CD40L^+^CD8^+^ T cells in a nonlymphopenic model. We previously generated a CD8^+^ T cell–specific CD40L^−/−^ mouse by breeding E8I-Cre mice (*24*) with CD40L^fl/fl^ mice (Fig. 3A). E8I-Cre × CD40L^fl/fl^ mice demonstrated a defect in CD40L expression restricted to mature CD8^+^ T cells, while CD40L expression in CD4^+^ T cells was not affected as shown after antigen-specific restimulation of splenocytes from ovalbumin-secreting *Listeria monocytogenes*–immunized mice (fig. S5). E8I-Cre × CD40L^fl/fl^ mice exhibited a profoundly impaired tumor control (19 of 26 mice) as compared to control CD40L^fl/fl^ mice (32 of 32 mice) and control E8I-Cre mice (10 of 10 mice) after cancer cell challenge (Fig. 3B). Although TAg-specific CD8^+^ T cells from E8I-Cre × CD40L^fl/fl^ lacked CD40L expression, the level of TAg-specific interferon-γ (IFN-γ)–producing CD8^+^ T cells was comparable between E8I-Cre × CD40L^fl/fl^ and WT mice (Fig. 3, C and D). These results, resulting from the depletion of a single factor in CD8^+^ T cells, underscore the tumoricidal potential of CD8^+^ T cell–derived CD40L even in a nonlymphopenic setting.

### CD40 expression on host cells is not required for rejection

**Fig. 4.**
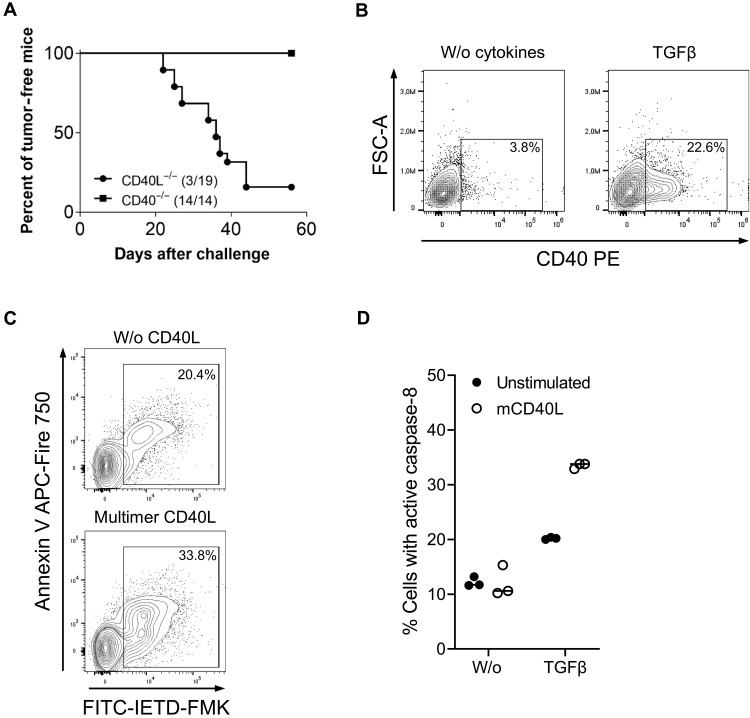
Role of CD40 expression on host and 9.27 cancer cells for tumor rejection. (**A**) CD40L^−/−^ and CD40^−/-^ mice were injected subcutaneously with 1 × 10^6^ 9.27 cancer cells. Summary of the tumor formation data obtained from two individual experiments, each with 6 to 10 mice per group. Tumor was defined as established when its volume reached >500 mm^3^. FSC-A, forward-scatter-area. (**B**) Cell surface expression of CD40 was analyzed after culturing 9.27 cancer cells, supplemented with or without TGFβ for 24 hours in three individual experiments. (**C** and **D**) The 9.27 cancer cells were treated for 24 hours with TGFβ and subsequently stimulated for further 24 hours with multimeric mouse CD40L. Thereafter, caspase-8 activity was determined with the fluorescence marker FITC-IETD-FMK and costained with annexin V. (C) The representative dot plots show the gating of annexin V and FITC-IETD-FMK–stained cancer cells after triggering CD40. (D) The diagrams summarize the frequencies of active caspase-8^+^ cancer cells, measured as triplicates of one of three representative experiments.

CD40L expressed by CD8^+^ T cells may either engage in the traditional cross-talk with host CD40^+^ antigen-presenting cells to enhance antitumor immunity or induce CD40-dependent noncanonical cytotoxicity in CD40^+^ cancer cells. To first evaluate the role of CD40 expressed by host cells, we challenged mice lacking CD40 or CD40L with TAg^+^ cancer cells. In case the tumor defense relies on the activation of CD40^+^ host cells such as antigen-presenting cells by CD40L-expressing T cells, the tumor growth rates should be comparable in CD40 or CD40L knockout (KO) mouse strains. However, 84% of the CD40L^−/−^ mice developed tumors (16 of 19 mice), whereas all CD40^−/−^ mice successfully prevented tumor formation (Fig. 4A). This reveals that the presence of CD40 on host antigen-presenting cells and/or tumor stromal cells is not essential for the successful rejection of the TAg^+^ cancer cells. Thus, we hypothesize that CD8^+^ T cells recognize tumor antigens presented by the cancer cells via MHC-I, whereupon they become activated and express CD40L, which directly triggers noncanonical cytotoxicity in CD40^+^ cancer cells.

### CD40-mediated noncanonical cytotoxicity activates caspase-8 resulting in cancer cell death

Hence, we next examined CD40 expression on the TAg^+^ cancer cells. In untreated cultures, CD40 expression on TAg^+^ cancer cells was almost absent but increased following incubation with transforming growth factor–β (TGFβ) (Fig. 4B), a cytokine that is frequently found in tumors and observed to be elevated in LoxPTAg mice that developed tumors (*25*, *26*). Consequently, CD40 expression was significantly increased on TAg^+^ cancer cells freshly isolated from tumors grown in lymphodepleted mice (fig. S6A). Upon restimulation of sorted TAg^+^ cancer cells based on CD40 expression, we observed that CD40 expression on TAg^+^ cancer cells is a dynamic process that is activated in most cells (fig. S6B). To investigate whether activating CD40 in TAg^+^ cancer cells directly induces cell death, as described for human carcinoma cells (*27*, *28*), we stimulated TGFβ pretreated and untreated cancer cells with multimeric CD40L. This approach mimics the homotrimeric CD40L found on the T cell membrane (*29*). CD40 triggering on cancer cells activated caspase-8, the initiator caspase of extrinsic apoptosis, assessed by the cell-permeable fluorescent marker FITC-IETD-FMK (Fig. 4, C and D). The activation of caspase-8 was increased in TGFβ pretreated samples that expressed sufficient CD40 levels (Fig. 4D). These observations corroborate that the engagement of CD40 on cancer cells facilitates CD40L-CD40–induced cytotoxicity.

### Overexpression of CD40 sensitizes TRAMP-C1 cancer cells to rejection by CD40L-expressing CD8^+^ T cell

**Fig. 5.**
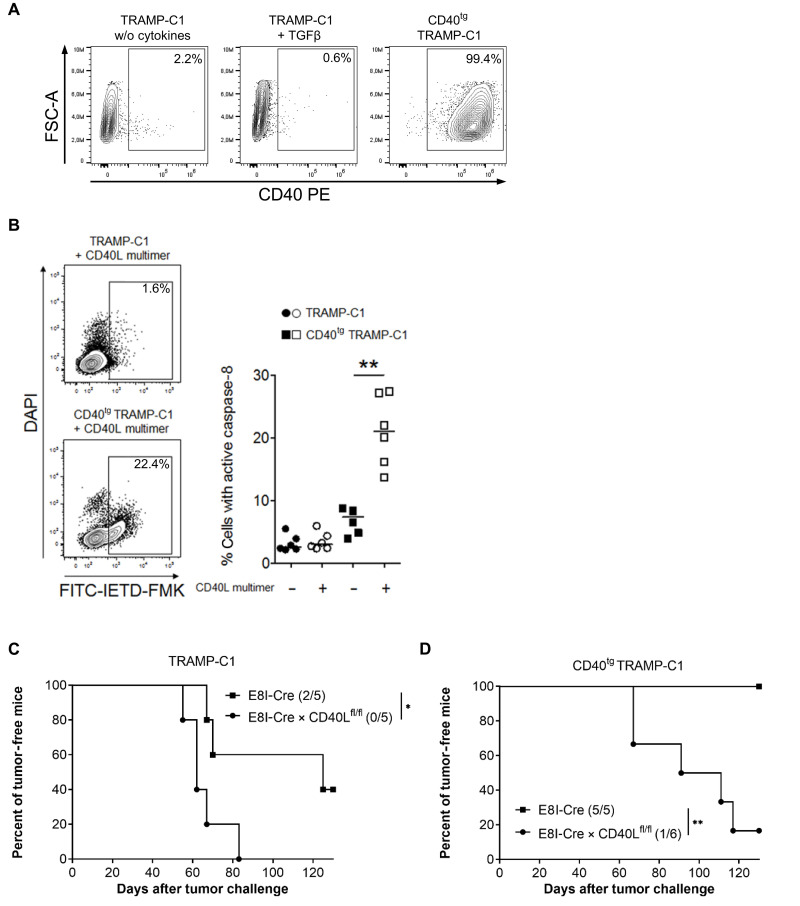
CD8^+^ T cell–mediated CD40 signaling in cancer cells prevents tumor formation. (**A**) Cell surface expression of CD40 was analyzed after culturing TRAMP-C1 and CD40^tg^ TRAMP-C1 cells with or without TGFβ for 24 hours. (**B**) Both TRAMP-C1 cancer cell lines were stimulated for 24 hours with multimeric CD40L. Thereafter, caspase-8 activity was determined with the fluorescence marker FITC-IETD-FMK. The representative dot plots show the gating of 4′,6-diamidino-2-phenylindole (DAPI) and FITC-IETD-FMK–stained cancer cells after triggering CD40. The diagrams summarize the frequencies of active caspase-8^+^ cancer cells. (**C** and **D**) E8I-Cre × CD40L^fl/fl^ and E8I-Cre as WT control mice were injected subcutaneously with 5 × 10^6^ TRAMP-C1 (C) or CD40^tg^ TRAMP-C1 (D) cancer cells. In the diagrams [(C) and (D)], the data from one of two representative experiments are shown. Per group, five or six mice were challenged. Tumor was defined as established when its volume reached >500 mm^3^. Statistical analysis: (B) Mann-Whitney *U* test: **P < 0.01 and [(C) and (D)] log-rank test: *P < 0.05 and **P < 0.01.

After demonstrating that CD40L^+^CD8^+^ T cells target CD40^+^TAg^+^ cancer cells directly, inducing cell death through a CD40L-CD40 interaction, we sought to extend our investigation to another in vivo cancer cell model. To this end, we used the immunogenic TRAMP-C1 progressor prostate carcinoma cell line, which harbors a distinct mutated neoepitope of the SPAS-1 protein recognized by CD8^+^ T cells (*30*). This was aimed to determine whether a strong contrast of CD40 expression has an impact on the tumor progression in WT and CD8^+^ T cell–specific CD40L^−/−^ mice. WT TRAMP-C1 cells express only neglectable levels of CD40, even after stimulation with TGFβ (Fig. 5A). A stable CD40-expressing TRAMP-C1 variant (CD40^tg^ TRAMP-C1) was generated by lentiviral transduction of the CD40 isoform I (Fig. 5A). In vitro stimulation of CD40^tg^ TRAMP-C1 cancer cells with multimeric CD40L led to a more than 10-fold increase in caspase-8 activity. This confirms our results obtained with TAg^+^ cells that CD40 signaling transmits an apoptotic signal in CD40^tg^ TRAMP-C1 cancer cells (Fig. 5B). Control E8I-Cre mice and E8I-Cre × CD40L^fl/fl^ mice were subsequently challenged with WT TRAMP-C1 and CD40^tg^ TRAMP-C1 cancer cells. In the E8I-Cre WT control group, half of the mice developed WT TRAMP-C1 tumors, yet all WT mice successfully rejected the CD40^tg^ TRAMP-C1 cells (Fig. 5, C and D). Conversely, all but one mouse in the E8I-Cre × CD40L^fl/fl^ group developed tumors, regardless of whether the cancer cells expressed CD40. This difference underscores the essential role of CD40L from CD8^+^ T cells in tumor immunity.

### CD40L from CD8^+^ T cells triggers noncanonical cytotoxicity in human CD40^+^ cancer cells

**Fig. 6.**
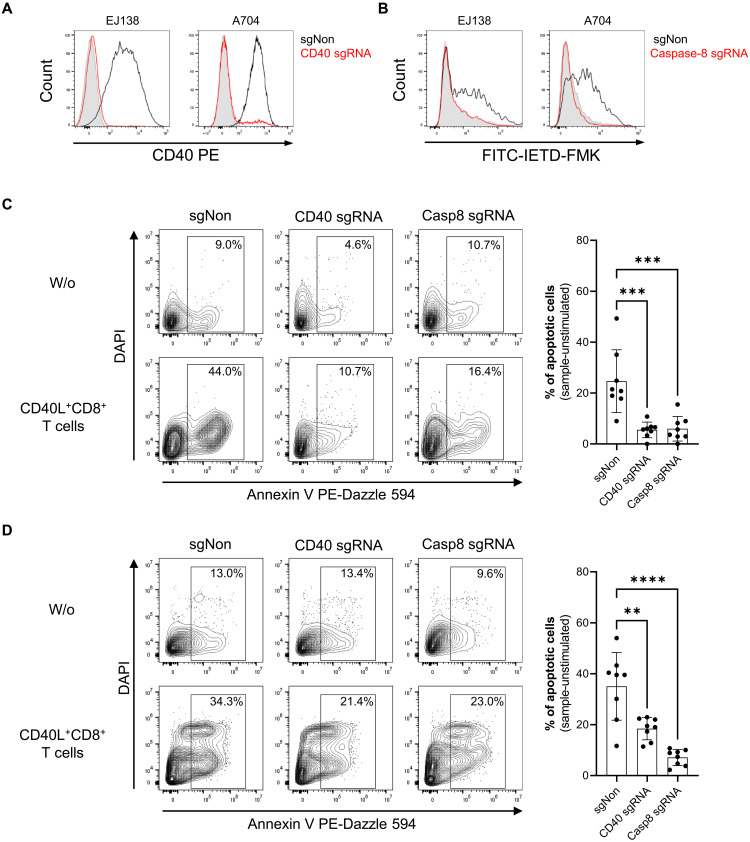
CD40L^+^CD8^+^ T cells mediate cell death in human CD40^+^ carcinoma cell lines by caspase-8 activation. (**A**) Histograms represent the CD40 expression on EJ138 or A704 transfected with nontargeting single guide RNA (black, sgNon) or cells depleted for CD40 by CRISPR-Cas9 and sgRNA against human CD40 (red). Gray-filled histograms show isotype staining. (**B**) sgNon (black) or caspase-8 sgRNA–treated (red) EJ138 and A704 cells were treated with multimeric CD40L for 24 hours, and caspase-8 activation was monitored by FITC-IETD-FMK staining. Gray-filled histograms represent the basal caspase-8 activation of unstimulated WT cells. (**C** and **D**) sgNon, CD40 sgRNA-, or caspase-8 (Casp8) sgRNA–treated variants of EJ138 (C) or A704 (D) were cocultivated with CD40L-enriched CD8^+^ T cells for 24 hours, and apoptosis was detected by annexin V and DAPI staining. Shown are representative dot plots with frequencies of technical triplicates (left) and bar graphs summarizing the data from experiments with eight different T cell donors. Statistical analysis: [(C) and (D)] Repeated measures ANOVA, followed by Dunnett’s test: **P < 0.01, ***P < 0.001, and ****P < 0.0001.

Next, we explored whether CD8^+^ T cell–derived CD40L-CD40 signaling similarly triggers cytotoxicity in human cancer cells through caspase-8 activation. To control the specificity of CD40-mediated and caspase-8–dependent cell death in an in vitro assay with CD8^+^ T cells, human EJ138 bladder carcinoma cells, and A704 renal adenocarcinoma cells, we generated CD40^−/−^ and caspase-8^−/−^ variants of both lines (Fig. 6, A and B). Deletion of either CD40 or caspase-8 markedly decreased cell death elicited by CD40L^+^CD8^+^ T cells (Fig. 6, C and D). Notably, eliminating caspase-8 resulted in a reduction of annexin V staining that was almost the same as that observed with CD40 KO. These results substantiate that CD8^+^ T cell–derived CD40L induces noncanonical cytotoxicity by activating the apoptosis-initiating caspase-8 in human cancer cells that express CD40.

### Resistance to CD40-mediated cytotoxicity is associated with poor overall survival in RCC

**Fig. 7.**
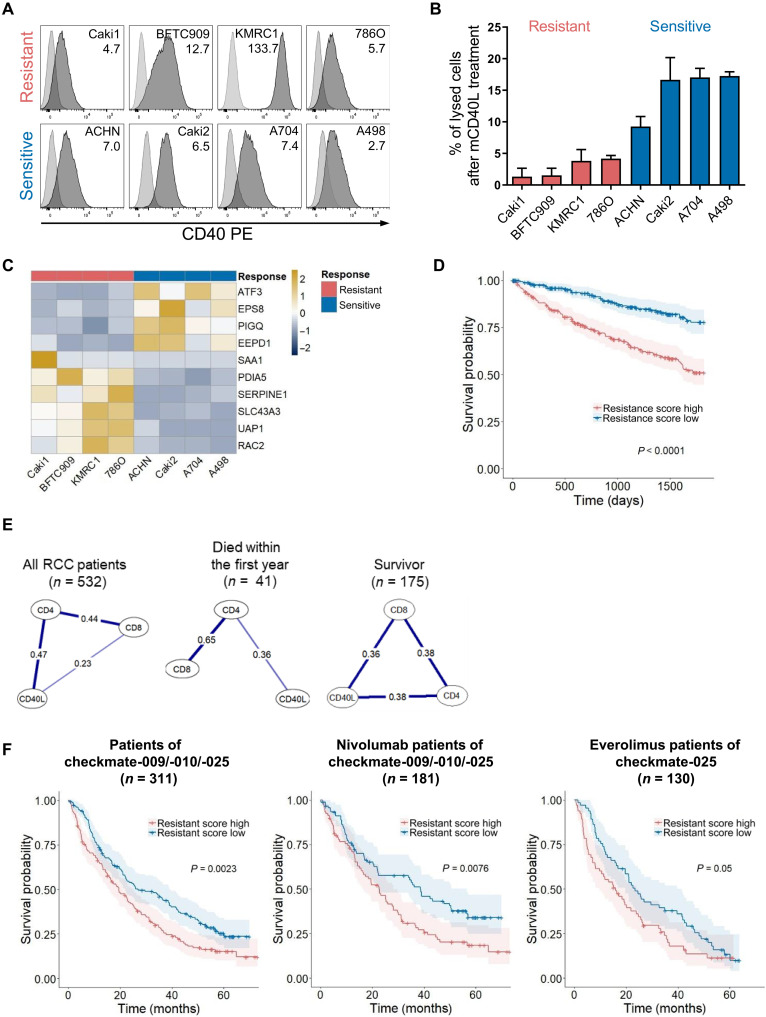
Resistance pattern for CD40-mediated cell death and correlations between CD8 and CD40L in different RCC cohorts. (**A**) In the histograms, the dark gray areas display the CD40 expression, and the light gray areas display the corresponding isotype control on eight different RCC lines. The included numbers represent the mean fluorescence intensity fold change between CD40 and isotype control staining. (**B**) Percentages of CD40L-induced lysis per RCC line after stimulation for 48 hours are plotted. Representative lysis values after backgorund substraction of each cell line of at least three individual experiments are depicted. (**C**) The heatmap represents the top 10 differentially expressed genes between CD40-resistant and CD40-sensitive RCC cell lines. (**D**) In the Kaplan-Meier survival plot, the resistance score of low and high groups of the TCGA-KIRC patient cohort is compared with each other. (**E**) Partial correlation networks of different patient groups are displayed, and the numbers are the partial correlation coefficients. (**F**) In the Kaplan-Meier survival plots, the resistance score of low and high groups of the cohorts is compared with all patients treated within the checkmate-009, checkmate-010, and checkmate-025 studies (left) or divided into two arms of the checkmate-025 study (middle nivolumab arm and right everolimus arm). Statistical analysis: (C) Described in Materials and Methods; [(D) and (F)] log-rank test.

We next evaluated CD40L-mediated cytotoxicity in patients with RCC. We chose RCC as the human tumor model because it not only exhibits high levels of CD8^+^ T cell infiltration (*31*) but also, according to The Cancer Genome Atlas (TCGA) Pan-Cancer dataset, displays the highest CD40 expression among all tumor types, excluding those occurring in hematopoietic and thymic tissues. Consistent with the reported data (*32*), we detected CD40 expression on all of eight tested RCC cell lines using flow cytometry (Fig. 7A). Subsequently, the RCC cell lines were incubated for 48 hours with multimeric CD40L before cell death was assessed using a lactate dehydrogenase (LDH) release assay (Fig. 7B). CD40L-CD40 signaling mediated by multimeric CD40L-induced cell death among the eight RCC cell lines varied, ranging from 1 to 20%. There was no direct correlation between CD40 expression density and CD40L-CD40 signaling–induced cell lysis; for instance, KMRC1, which had the highest CD40 expression, exhibited 78% less CD40-mediated cell death compared to A498, which had the lowest level of CD40 expression. On the basis of the extent of cell death induced, we categorized the four lines with the lowest death rates (1 to 4%) as resistant and the other four lines (8 to 20%) as sensitive to CD40L-CD40 signaling–mediated cytotoxicity. We then compared the gene expression profiles of both groups using RNA sequencing data from the Cancer Cell Line Encyclopedia (CCLE) (*33*). Among the top 10 most significantly and differentially expressed genes were six genes (*SAA1*, *PDIA5*, *SERPINE1*, *SLC43A3*, *UAP1*, and *RAC2*), which were up-regulated in the resistant cell lines (Fig. 7C). To assess the contribution of these six identified genes to resistance against CD40L-CD40–mediated noncanonical cytotoxicity, we conducted targeted CRISPR-Cas9 gene KOs on the three genes that were most consistently and highly expressed across the resistant cell lines. Accordingly, the depletion of either *RAC2*, *SERPINE1*, or *SLC43A3* was able to sensitize previously resistant 786O, BFTC909, and KMRC1 cells to cell death mediated by the multimeric CD40L, proportional to the respective gene expression in WT cells (fig. S7, A to C).

To investigate whether the gene expression pattern linked to resistance against CD40L-mediated noncanonical cytotoxicity holds significance in patients with RCC, we used the mean expression values of these genes to compute a resistance score. According to this score, we classified patients of the TCGA–kidney renal clear cell carcinoma (KIRC) cohort into groups with a low or high resistance score, dependent on whether their individual resistance score for CD40L-mediated cell death was below or above the median within the cohort. Survival analysis revealed that patients with a low resistance score exhibited significantly better survival rates compared to those with a high resistance score (*P* = 2 × 10^−9^) (Fig. 7D). Correspondingly, the resistance scores progressively increased from stages I to IV of RCC tumor progression, suggesting that the ability to evade CD40L-CD40–mediated cytotoxicity is a critical factor for tumor progression and cancer immune evasion (fig. S8).

To test the contribution of CD40L expressed on CD8^+^ rather than CD4^+^ T cells, that in humans, the CD40L noncanonical cytotoxicity–mediating signal originates specifically from CD8^+^ rather than CD4^+^ T cells, we performed a partial correlation analysis on the expression of CD8 (mean of *CD8A* and *CD8B*), CD4, and CD40L (*CD40LG*) across the entire TCGA-KIRC cohort (Fig. 7E). This approach was chosen as we expected high levels of covariance between these immunological hallmark genes, which would dominate the results by standard correlation analysis. As expected, in the complete TCGA patient cohort, the expression of *CD40LG* correlated stronger with that of CD4 as with CD8 (including both *CD8A* and *CD8B*). However, analyzing the correlation in patients who passed away within the first year versus those reported as survivors revealed a decrease in both *CD40LG* and CD8 expression correlation among early fatalities but an increase among survivors. These observations strongly suggest that CD40L derived from CD8^+^ T cells, coupled with a low resistance score, contributes positively to the overall survival of patients with RCC.

To corroborate the RCC’s CD40L-CD40–mediated susceptibility to noncanonical cytotoxicity in a different patient cohort and to confirm the pivotal role of CD8^+^ T cell–derived CD40L, we used data from the checkmate-009, checkmate-010, and checkmate-025 studies, which were compiled in the study of Braun *et al*. (*34*). In these clinical trials, patients with stage IV metastatic RCC were treated with the anti-PD1 blocking antibody nivolumab or with the mechanistic target of rapamycin inhibitor everolimus. Analyzing tumor RNA sequencing data from all checkmate patients showed that elevated expression of the six genes previously identified, which are associated with resistance to CD40L-CD40 signaling–induced noncanonical cytotoxicity, is linked to worse survival outcomes (Fig. 7F). When dissecting the data from patients treated with everolimus and nivolumab, the survival disparities were primarily observed in those receiving PD1 inhibitor. This suggests that CD40L-CD40–mediated cytotoxicity is most pronounced when T cell responses, likely including T cell–derived CD40L, are augmented by immune checkpoint inhibition. Comparable to the TCGA cohort, partial correlation analyses of nivolumab-treated patients suggest the presence of CD40L-expressing CD8^+^ T cells in tumors of patients who survived at least 1-year posttherapy (fig. S9). Thus, our analysis reveals that tumors infiltrated with higher frequencies of CD40L-expressing CD8^+^ T cells are better positioned to leverage the cancer’s vulnerability to CD40-mediated noncanonical cytotoxicity.

## DISCUSSION

CD8^+^ T cells play an indispensable role in antitumor immunity, and their presence within tumor environments is consistently associated with a favorable prognosis (*35*, *36*). Activated tumor-specific CD8^+^ T cells are able to directly target CD40-expressing cancer cells within the tumor microenvironment, provided that these cells display tumor antigens. Following antigen activation, CD8^+^ T cells unleash a plethora of effector molecules, including cytokines (e.g., IFN-γ and TNFα) and cytotoxic agents inducing cell death (e.g., granzymes and FasL), which are integral to the process of tumor rejection (*22*, *37*–*39*). In this study, we identified CD40L expression by CD8^+^ T cells as a crucial, unexpected, and therefore noncanonical function in the cellular antitumor response. While antitumor effects of the CD40L-CD40 axis in dendritic cell activation by CD4^+^ T cells have been documented (*4*–*7*, *40*, *41*), its ability to remodel the tumor microenvironment has previously been studied (*42*, *43*). Our research now demonstrates that CD40L on CD8^+^ T cells halts tumor progression by directly triggering caspase-8 activation in CD40^+^ cancer cells, resulting in CD40L-mediated cytotoxicity.

Insights from previous research suggest that targeting the CD40L-CD40 pathway could offer therapeutic benefits for a wide range of patients, given the prevalent CD40 expression in various cancers such as melanoma and carcinoma, which are vulnerable to CD40-triggered cell death (*13*, *14*). In this context, we hypothesize that tumor-specific CD40L-expressing CD8^+^ T cells could enhance tumor-rejecting immune responses. Unlike CD4^+^ T cells, which operate under MHC-II restriction, CD8^+^ T cells can activate CD40 on virtually any cell, including cancer cells, upon recognizing specific peptide/MHC-I complexes with their T cell receptor (TCR).

A key drawback of current agonistic anti-CD40 antibodies is their indiscriminate targeting of all CD40^+^ cells, likely contributing to their substantial toxicities (*44*). Serious side effects have been reported from clinical use, including liver damage, inflammatory eye disorders, thrombocytopenia, and cytokine release syndrome (*45*, *46*). Furthermore, fatal outcomes from combining anti-CD40 antibodies with chemotherapy were documented in animal studies (*47*). In contrast to anti-CD40 antibodies, CD40L is specifically induced in CD8^+^ T cells upon tumor-specific antigen recognition, making them precise cellular CD40 agonists. This targeted activation and the natural trimeric form of CD40L on T cells may enhance antitumor efficacy, offering a more potent and targeted cytotoxic response against cancer cells (*18*).

We identified a six-gene signature in RCC cell lines indicative of resistance to CD40-mediated cytotoxicity, offering a potential stratification strategy to predict patient survival and responsiveness to T cell–based immunotherapies. Notably, gene silencing of *RAC2*, *SERPINE1*, and *SLC43A3*, which were described to be regulated by TNFSF stimulation (*48*–*51*) and as counter regulators of apoptosis (*52*–*55*), effectively enables CD40-induced cell death in previously resistant cells. Consistent with our findings, a previous report demonstrates that CD40 expression on RCC and increased CD8^+^ T cell infiltration are associated with prolonged patient survival (*56*). Our analysis also highlights that T cells, (re-)activated through immune checkpoint blockade therapy, can effectively target CD40^+^ cancer cells, exploiting their CD40 susceptibility.

While CD40 is expressed in various cancers such as carcinomas, melanomas, and B cell lymphomas, its engagement does not uniformly lead to cancer cell death. The outcome of CD40 signaling is subject to variation, influenced by the nature of CD40L (whether soluble or membrane-bound), signal intensity, specific cancer cell pathway modifications (*27*), and, now, also the cellular origin of CD40L. For instance, in conditions such as follicular lymphoma, CD40 signaling promotes survival rather than cell death (*57*, *58*).

Equipping T cell products with the capacity to express CD40L could augment their therapeutic efficacy, as demonstrated in a recent preclinical study of T cells constitutively expressing CD40L and a chimeric antigen receptor (*59*). However, continuous CD40L expression could pose risks akin to those of systemic anti-CD40 antibodies. Therefore, promoting natural, TCR-activated CD40L expression in T cell therapies may minimize adverse effects and improve specificity. Last, enhancing the presence of CD40L^+^CD8^+^ T cells or sensitizing cancer cells to CD40-induced cytotoxicity with drugs could serve as effective strategies to use this additional antitumor mechanism.

## MATERIALS AND METHODS

### Mice and cell preparation

C57BL/6J mice were purchased from Charles River Laboratories and C57BL/6-RAG1^−/−^ (RAG1^−/−^) from Taconic. The following mice were obtained from the Jackson Laboratory and bred and housed under specific pathogen–free conditions at the institution’s animal facility (Charité): CD40L^−/−^ (RRID: IMSR_JAX:002428), CD4^−/−^ (RRID: IMSR_JAX:002663), CD8^−/−^ (RRID: IMSR_JAX:002665), CD45.1 (RRID: IMSR_JAX:002014), and CD40^−/−^ (RRID: IMSR_JAX:002928).

Mice from both sexes were challenged at 6 to 12 weeks of age and euthanized at indicated time points. All animal experiments were performed in accordance with the German laws and permission from the ethics board of the Charité.

Single-cell suspensions were obtained from the spleens or lymph nodes and were cultured as the murine cancer cell lines 9.27 and 16.113 in RPMI 1640 medium supplemented with penicillin (100 U/ml), streptomycin (0.1 mg/ml), glutamine (0.3 mg/ml), 10% heat-inactivated fetal bovine serum (FBS), and 50 μM β-mercaptoethanol. All animal experiments were conducted in accordance with German laws and the authorization of the local authority—LaGeSo—under registration numbers G0163/10, G0243/12, and G0307/13.

### Generation and testing of E8I-Cre × CD40L^fl/fl^ mice

For the generation of CD40L *^fl/f^* mice, a targeting vector was constructed that contains a genomic fragment of the mouse CD40L gene with exons 3 and 4 flanked by LoxP sites and neomycin and puromycin resistance as selection markers. The linearized targeting vector was introduced into C57BL/6NTac (RRID: IMSR_TAC:B6) embryonic stem (ES) cells via electroporation. Puromycin and G418 resistant clones were counter-selected with ganciclovir, and correct integration was analyzed via Southern blot. Targeted C57BL/6NTac ES cells were injected into Balb/c blastocysts, and germline transmission was achieved by crossing chimeric mice with C57BL/6J. Mice with targeted mutation in the germ line were crossed with Flp-deleter mice to remove neomycin and puromycin resistance genes. CD40L *^fl/fl^* mice were first backcrossed on C57Bl6 for 10 generations before crossing with E8I-Cre mice [C57BL/6-Tg(Cd8a-cre)1Itan/J (*24*), provided by I. Taniuchi, Yokohoma] to generate a CD8^+^ T cell–specific deletion of CD40L. Male mice were challenged at 6 to 12 weeks of age.

To prove the specificity of the Cre recombinase expression E8I-Cre and E8I-Cre × CD40L^fl/fl^, mice were immunized by injection into the tail veins of a sublethal dosage of 2000 *L*. *monocytogenes*–secreting ovalbumin. After 7 days, splenocytes were isolated and stimulated with the MHC-I–restricted ovalbumin peptide SIINFEKL or with recombinant ovalbumin for 6 hours in the presence of brefeldin A. Staining and fluorescence-activated cell sorting (FACS) measurement were performed as described below.

### Antibodies

The following antibodies (clones) conjugated to fluorescein isothiocyanate (FITC), phycoerythrin (PE), PerCP, allophycocyanin (APC), AF700, APC-Cy7, PE-Cy7, PacBlue, or BV421 were purchased from the listed companies. BD Biosciences: αmCD4 (RM4-5), αmCD8 (53-6.7), and αhIFN-γ (B27); Miltenyi Biotec: αmCD40L (MR1) and αmCD40 (FGK 45.5); eBioscience: αmCD4 (GK1.5), αmIFN-γ (XMG1.2), and αhIL-2 (N7.48A); BioLegend: αmCD3 (145-2C11), αhCD3 (UCHT-1), αmIL-2 (JES6-5H4), αmTNFα (MP6-XT22), αmCD45.1 (A20), αmCD326 (G8.8), αmCD90.2 (53-2.1), αmCD31 (W18222B), αhCCR7 (G043 H7), αhCD45RA (HI100), αhCD4 (RPA-T4), αhCD8 (RPA-T8, SK1), αhCD40L (24-3-1), and αhTNFα (Mab11). To avoid Fc receptor binding, murine cells were stained in the presence of αmFcγ receptor (2 μg/ml; 2.4G2), and human cells were stained with Beriglobin (1 mg/ml; Sanofi-Aventis).

### Cancer cell challenges

To induce tumors, in vitro–passaged 1 × 10^6^ 9.27 or 5 × 10^6^ TRAMP-C1 (RRID: CVCL_3614) cancer cells were injected subcutaneously into the mice. In the TAg-specific T cell response kinetic experiment, 1 × 10^7^ 16.113 cancer cells were injected intraperitoneally. Both SV40 TAg^+^ cancer lines were derived from SV40 LoxPTAg mice (*23*), and the TRAMP-C1 cancer cell line was purchased from American Type Culture Collection. All cancer cell lines were tested routinely by polymerase chain reaction (PCR) for mycoplasma contamination (Merck Millipore). The tumor volumes were determined by a blinded experimenter at least twice a week using a vernier calliper (Knighton tools) and were calculated using the formula for hemiellipsoid volume = *lwh*(1/2).

For the transfer in RAG1^−/−^, CD8^+^, and CD4^+^ T cells from C57BL/6 (or CD45.1) or CD40L^−/−^ mice, T cells from splenocytes were isolated by magnetic microbeads (Miltenyi Biotec). A total of 1 × 10^6^ T cells per mouse were injected intravenously on the same day as the injection of 9.27 cancer cells.

### Flow cytometry, cell sorting, stimulation, and T cell count

Enrichment of mouse T cells was performed using microbeads (Miltenyi Biotec). T cell purity of >95% was confirmed by flow cytometry for all transferred T cell populations. For the sorting and analysis of live cells, 400 nM 4′,6-diamidino-2-phenylindole (DAPI; Molecular Probes) was added. For the analysis of antigen-specific T cells, 5 × 10^6^ splenocytes were stimulated for 6 hours at 37°C with peptide I (1 μg/ml; SAINNYAQKL) and/or peptide IV (VVYDFLKL) from SV40 TAg (JPT) in the presence of brefeldin A (2 μg/ml; Sigma-Aldrich) for the assessment of CD40L and cytokines (*60*). Intracellular cytokine and CD40L staining was performed for 30 min after fixation and permeabilization with BD FACS Lysing and BD FACS Permeabilizing Solution 2 (BD Biosciences). The cells were subsequently analyzed using a LSRII flow cytometer (BD Biosciences).

CD40 expression was analyzed after culturing 1 × 10^5^ 9.27 cancer cells for 24 hours with or without recombinant human TGFβ (10 ng/ml; BioLegend). Subsequently, CD40 was stained extracellularly for 10 min, and cells were analyzed on a CytoFLEX LX (Beckman Coulter). To evaluate CD40 dynamics, stimulated cells were sorted on the basis of CD40 expression on a BD FACSAria II (BD). Sorted CD40-positive and CD40-negative cells were cultured for 10 days before restimulation with TGFβ (10 ng/ml) and subsequent CD40 detection.

To assess CD40 expression on TAg^+^ cancer cells in vivo, 1 × 10^6^ 9.27 cells were injected in lymphodepleted C57BL/Rag1 KO mice (RRID: IMSR_JAX:034159, the Jackson Laboratory) to prevent immediate rejection of CD40^+^ cancer cells. After 16 days, single-cell suspensions from tumors were generated by mechanical dissociation and filtering through 70-μm MACS SmartStrainers (Miltenyi Biotec). CD40 was analyzed by flow cytometry on viable EpCAM^+^ cancer cells not expressing CD31 (fibroblasts), CD90.2 (endothelial cells), or CD45.1 (immune cells).

Blood T cell counts were measured on a MACSQuant flow cytometer (Miltenyi Biotec) after lysing erythrocytes (Buffer EL, QIAGEN) and staining the remaining leukocytes.

### In vivo cytotoxicity assays

SV40 TAg–specific in vivo cytotoxicity assays for SV40 TAg peptide IV were performed, as previously described (*61*). A total of 1 × 10^7^ splenocytes/ml from C57BL/6 mice were loaded with peptide IV (1 μg/ml) for 15 min and thereafter labeled with 0.75 μM CFDA [high concentration of carboxyfluorescein diacetate succinimidyl ester (CFSE^high^)] for 15 min. Unloaded splenocytes were labeled with 0.075 μM CFDA (CFSE^low^). A total of 2 × 10^7^ mixed CFSE^high/low^ splenocytes at a 1:1 ratio were injected intravenously at day 28 after the tumor challenge. After 18 hours, CFSE-labeled cells in the spleen of recipient mice were analyzed by flow cytometry. Specific killing was calculated as [1 − (ratio of control mouse/ratio of immunized mouse)] × 100, where the ratio is defined as CFSE^low^ percentage/CFSE^high^ percentage.

### Caspase-8 assays and viable cell counts

For the detection of caspase-8 activity upon CD40 ligation, a total of 1 × 10^6^ murine cancer cells were plated in T25 flasks in RPMI 1640 supplemented with 5% FBS (Merck), penicillin (100 U/ml), and streptomycin (0.1 mg/ml; Sigma-Aldrich). To induce CD40 on 9.27 cells, cells were cultured with murine TGFβ (10 ng/ml; BioLegend) at 37°C and 5% CO_2_. After 24 hours, 5 × 10^4^ unstimulated or prestimulated cells were seeded in 96-well flat-bottom plates and treated with multimeric mouse CD40L (1 μg/ml; AdipoGen) or left untreated for further 24 hours. Detection of active caspase-8 in cultures was performed with the CaspGLOW Fluorescein Active Caspase-8 Staining Kit (Invitrogen). Briefly, after 24-hour incubation, adherent murine or human cancer cells were gently detached and harvested using TrypLE (Gibco). After one washing step, cells were stained with the FITC-labeled caspase-8 inhibitor IETD-FMK for 15 min. Where indicated, cells were additionally stained with annexin V (BioLegend) and/or 400 μM DAPI (Molecular Probes). After staining, cells were immediately acquired on a CytoFLEX LX (Beckman Coulter).

To assess the cell death induction in WT, CD40 KO or caspase-8 KO EJ138 or A704 cells, 3 × 10^4^ cancer cells were cultured per well of a 96-well plate in Dulbecco’s modified Eagle’s medium (DMEM) supplemented with 1% FBS (Merck), penicillin (100 U/ml), and streptomycin (0.1 mg/ml; PAA).

After stimulation of CD8-enriched T cells from two healthy donors with phorbol 12-myristate 13-acetate (PMA; 10 ng/ml) and ionomycin (1 μg/ml; Sigma-Aldrich) for 5 hours at 37°C, viable CD3^+^CD8^+^CD40L^+^ lymphocytes were sorted on a FACS AriaII (BD). After sorting, T cells were expanded for 12 days in X-Vivo-15 medium (Lonza) supplemented with 5% human antibody serum (PAN-Biotech), IL-15 (50 ng/ml), and IL-7 (10 ng/ml; Miltenyi Biotec). After a 2-day resting period (medium depleted of cytokines), T cells were reactivated using the initial PMA/inomycin protocol, resulting in about 75% CD40L^+^CD8^+^ T cells, thoroughly washed three times, and then cocultured with EJ138 or A704 cell lines for 24 hours in a 1:3 (target:effector) ratio. Detection of active caspase-8 and apoptotic cells was performed as described above for murine cancer cells. Human blood was obtained from healthy volunteers after providing informed consent and in accordance with Declaration of Helsinki.

### Generation of KO cell lines using CRISPR-Cas9

To generate cells depleted for CD40, caspase-8, RAC2, SERPINE1, or SLC43A4, we seeded 1 × 10^5^ cells in 24-well plates in Opti-MEM Reduced serum medium (Gibco) for 24 hours. The next day, cells were treated with 7.5 pmol of TrueGuide Synthetic single guide RNA (sgRNA) against human *CD40* (ID: CRISPR714647), *caspase-8* (ID: CRISPR700788), *RAC2* (ID: CRISPR753080), *SERPINE1* (ID: CRISPR835056), *SLC43A3* (ID: CRISPR172985), or the TrueGuide sgRNA Negative Control nontargeting 1, together with TrueCut Cas9 Protein v2 and Lipofectamine CRISPRMax Transfection Reagent (all Invitrogen) according to the manufacturer’s protocol. After 3 days, cells were recovered and cultured in DMEM supplemented with 10% FBS (Merck), penicillin (100 U/ml), and streptomycin (0.1 mg/ml; PAA). Efficacy of CD40 KO was determined by flow cytometry. Depletion of caspase-8 and sensitivity to CD40-mediated cell death were analyzed after stimulation of generated cell lines with human CD40L multimer kit (10 μg/ml; Miltenyi Biotec) for 48 hours, and caspase-8 initiation was detected as described above.

### Generation of the CD40 transgenic TRAMP-C1 (CD40^tg^ TRAMP-C1)

For the generation of TRAMP-C1 cell lines stably expressing the murine CD40, murine B cells from a C57Bl6/J mouse were isolated for RNA extraction and cDNA transcription using reverse transcriptase (Thermo Fisher Scientific). The cDNA served as a template for PCR amplification of the CD40 isoform I sequence (forward primer: ATTACGCGTGCCACCATG-GTGTCTTTGCCTCGGCTGTGC; reverse primer: ATTACGCGTGCCACCATGGTGTCTTTGCCTCGGCTGTGC). The generated amplicon was cloned into the lentiviral vector pLenti cytomegalovirus green fluorescent protein Puro (658-5) (Addgene, plasmid #17448; gift from E. Campeau) modified by the insertion of an EF1α promoter upstream of the CD40 sequence. Together with the packaging plasmids pMD2.G (Addgene, plasmid #12259), pMDLg/pRRE (Addgene, plasmid #12251), and pRSV-Rev (Addgene, plasmid #12253; all three packaging plasmids were provided by D. Trono), the transgene vector pLenti_CD40 was transfected into human embryonic kidney 293T cells (RRID: CVCL_0063) by the standard transfection protocol using polyethylenimine (Polyscience Inc.), and supernatants containing the released lentiviruses were cleared by filtration and concentrated with Lenti-XTM Concentrator (Clontech). For subsequent transduction of TRAMP-C1 with generated lentiviruses, 1 × 10^5^ TRAMP-C1 cells were seeded in six-well plate and incubated at 37°C overnight prior transduction. A total of 1 × 10^6^ transducing unit in 1 ml of TRAMP-C1 medium and 4 μg of polybrene (Sigma-Aldrich) were added to TRAMP-C1 cells in each well, followed by a spinoculation step at 2000*g* and 30°C for 1 hour. Afterward, the medium was adjusted to 2 ml per well, and TRAMP-C1 cells were incubated at 37°C for 72 hours. Last, TRAMP-C1 cells were stained with αmCD40-APC (FGK 45.5; Miltenyi Biotec) for FACS to enrich CD40^+^ TRAMP-C1 cells.

### Resistance score determination and analysis of RCC patient cohorts

To test CD40 sensitivity in RCC, eight RCC cell lines [Caki1 (RRID: CVCL_0234), BFTC909 (RRID: CVCL_1084), KMRC1 (RRID: CVCL_2983), 786-O (RRID: CVCL_1051), ACHN (RRID: CVCL_1067), Caki2 (RRID: CVCL_0235), A704 (RRID: CVCL_1065), and A498 (RRID: CVCL_1056)] were tested for CD40 expression by flow cytometry. Subsequently, 5 × 10^4^ cancer cells were plated in 96-well flat-bottom plates in phenol red–free DMEM supplemented with 1% FBS (Merck), penicillin (100 U/ml), and streptomycin (0.1 mg/ml; PAA) and cultured with or without human multimeric CD40L (10 μg/ml; Miltenyi Biotec) for 48 hours. Induction of CD40L-mediated cell death was assessed by LDH release from cancer cells into the culture medium using the Cytotoxicity Detection Kit (Roche). For subsequent determination of differentially expressed genes, a cutoff of 5% specific lysis was applied to identify CD40-resistant cell lines calculated using the following formula

$$\left[\left({\text{LD}}_{\text{sample}}-{\text{LD}}_{\text{background}}\right)/\left({\text{LD}}_{\text{Triton}}-{\text{LD}}_{\text{background}}\right)\right]\times 100\%$$

The preprocessed gene expression data of the CCLE dataset for the sensitive and resistant RCC cancer lines were downloaded from Broad DepMap Portal (version: Expression Public 22Q1). For differential gene expression analysis between the RCC lines, we used the processing pipeline provided by the R package edgeR (V.3.38.4) together with limma R package (V.3.52.3), which uses between-sample normalization method (trimmed mean of *M* values), voom transformation, linear model fitting, and contrast matrix. Last, empirical Bayes shrinkage was performed to estimate *t* statistic and the related *P* values. The top 10 differentially expressed genes, all with *P* < 0.001, were selected and displayed as heatmap using the R package pheatmap (V.1.0.12). Because of the rather small number of cell lines in particular compared to the many analyzed genes, the false discovery rates of the selected genes were methodological relatively high and ranged between 0.2 and 0.5.

The RNA sequencing gene expression dataset of the TCGA-KIRC cohort was obtained from the UCSC Xena platform using the R package UCSCXenaTools (V.1.4.8) (*62*). The RNA sequencing data of the checkmate studies were directly downloaded from the supplementary information of the manuscript from Braun *et al.* (*34*).

To calculate the resistance score for CD40-mediated cancer cell killing, the mean values of the six genes that were elevated in the resistant cell lines were used. Afterward, we determined the median resistance score expression of the tested patient cohorts to distinguish patients with a low or high resistance score. The Kaplan-Meyer survival plots and the tumor stage versus resistant score box plot as well as the log-rank test were performed by using the R packages survival (V. 3.4.0), survminer (V. 0.4.9), and ggplot2 (V. 3.3.6).

For the partial correlation analysis, the cohorts were first grouped in patients who died within the first year and patients who were reported as survivors including patients that were censored for different reasons. The calculation and plotting were done with R package bootner (V.1.5) using the function estimateNetwork with the partial correlation algorithm EBICglasso that based on Gaussian Markov random field estimation using the graphical LASSO and extended Bayesian information criterion to select a regularization parameter.

### Statistics

Prism V.5 (GraphPad Inc.) or R version 4.0.0 was used for plotting and statistical analysis. The used statistical tests are specified in the figure legends.

### Ethics approval and consent to participate

Acquisition of blood samples and peripheral blood mononuclear cells from healthy volunteers was approved by the Ethics Committee of Charité–Universitätsmedizin Berlin (EA4/219/20). Informed consent from all study participants was obtained regarding the performed analyses.
