# Supplementary material for: CD8+ T cell–derived CD40L mediates noncanonical cytotoxicity in CD40-expressing cancer cells
Source: Sci Adv. 2025 May 21;11(21):eadr9331. doi: 10.1126/sciadv.adr9331 (PMC12094236; doi:10.1126/sciadv.adr9331)
Supplement: Supplementary file 1 — Figs. S1 to S9 [file sciadv.adr9331_sm.pdf]

Science Advances
AAAS

Supplementary Materials for

CD8$^+$ T cell–derived CD40L mediates noncanonical cytotoxicity in CD40-expressing cancer cells

Phillip Schiele *et al.*

Corresponding author: Phillip Schiele, phillip.schiele@charite.de; Marco Frentsch, marco.frentsch@bih-charite.de

Sci. Adv. **11**, eadr9331 (2025)
DOI: 10.1126/sciadv.adr9331

This PDF file includes:

Figs. S1 to S9

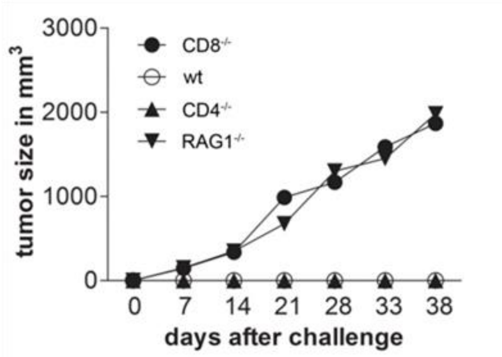

Supplementary Figure 1. $CD8^+$ T cells are required for the prevention of SV40 TAg^+ 9.27 cancer cell outgrowth. $RAG1^{-/-}$, $CD4^{-/-}$, $CD8^{-/-}$ and wt mice were injected s.c. with $1x10^6$ 9.27 cancer cells and tumor progression was monitored. Data represent the mean ± s.d of the tumor volume from one out of two representative experiments with three mice per strain.

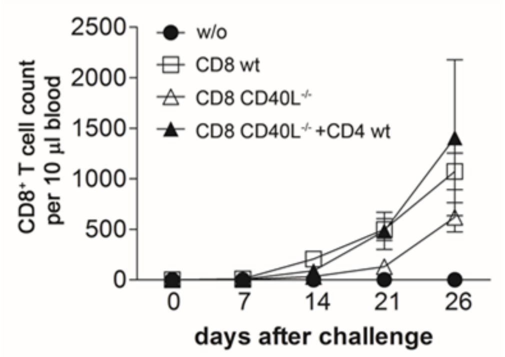

Supplementary Figure 2. $CD8^+$ T cell expansion in tumor-bearing $RAG1^{-/-}$ mice. $RAG1^{-/-}$ mice were injected s.c. with $1x10^6$ 9.27 cancer cells and at the same day treated i.v. with $1x10^6$ wt or $CD40L^{-/-}$ $CD8^+$ T cells with and without $1x10^6$ wt $CD4^+$ T cells. Blood $CD8^+$ T cell counts of each mouse were determined by flow cytometry (n = 4 or 5 mice per group). Data represent one experiment out of three.

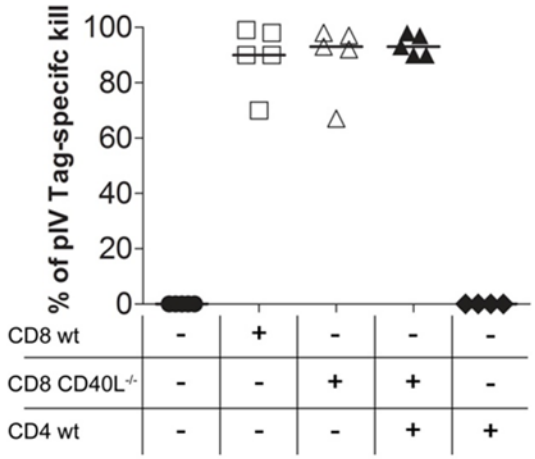

Supplementary Figure 3. Transferred CD40L$^{-/-}$ CD8^{+} T cells kill equally to wt CD8^{+} T cells in a TAg-specific *in vivo* cytotoxicity assay. 28 days after 9.27 cancer cell challenge and T cell transfer RAG1$^{-/-}$ mice were used for peptide IV TAg-specific *in vivo* cytotoxicity assay. Shown are individual mice and the median for TAg-specific kill (n = 4 or 5 mice per group). Data represent one experiment out of two.

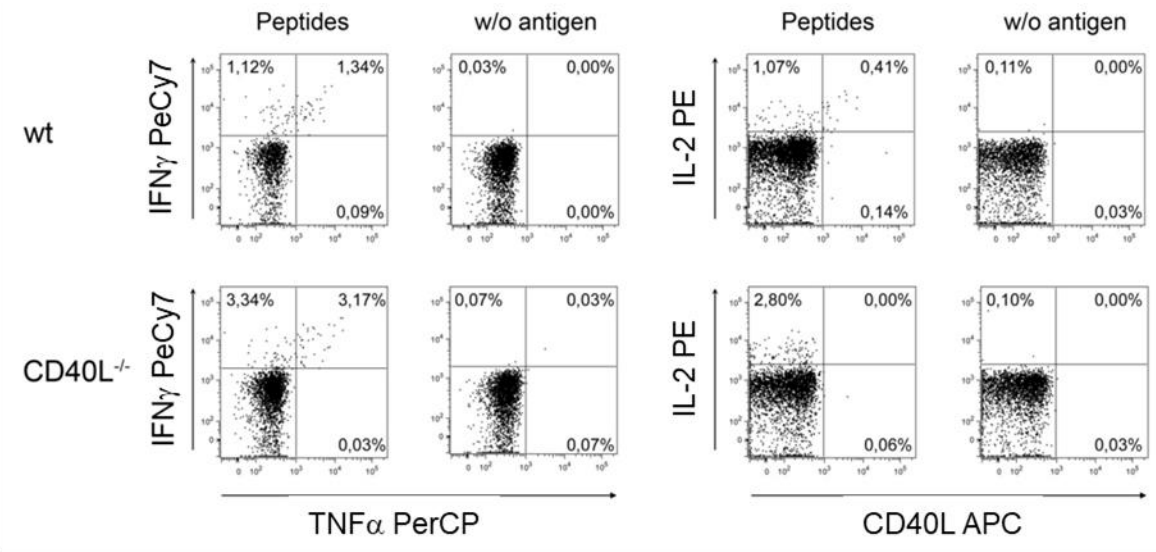

Supplementary Figure 4. Cytokine expression in SV40 TAg-specific CD8$^+$ T cells after 9.27 cancer cell challenge. At day 27, blood cells of RAG1$^{-/-}$ mice reconstituted with wt or CD40L$^{-/-}$ CD8$^+$ T cells were stimulated for 6h with SV40 TAg peptides I and IV. Intracellular cytokines and CD40L staining in CD3$^+$CD8$^+$ lymphocytes are shown as dot-plots from one representative mouse out of 4 or 5. Data represent one experiment out of two.

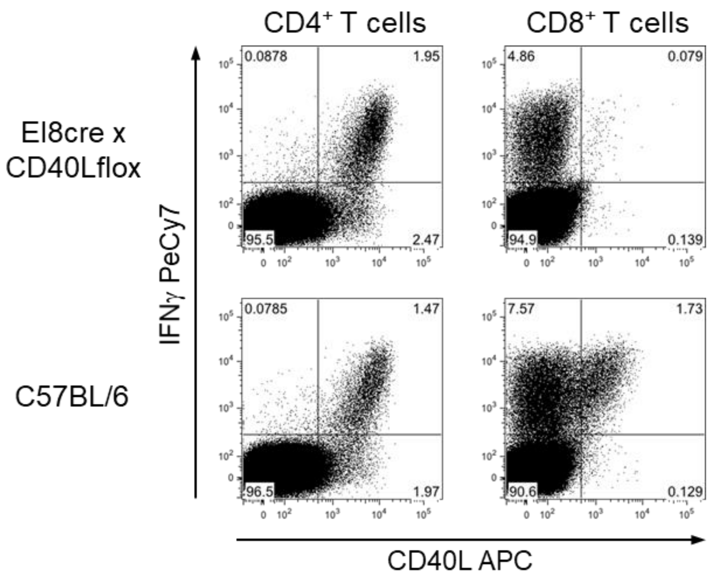

Supplementary Figure 5. CD4^{+} T cell-derived CD40L is not affected in E8I-Cre x CD40L$^{fl/fl}$ mice. E8I-Cre (wt) and E8I-Cre x CD40L$^{fl/fl}$ mice were i.v. injected with 2000 Listeria monocytogens secreting ovalbumin and 7 days later splenocytes were isolated and stimulated with the MHC-I restricted ovalbumin peptide SIINFEKL or with recombinant ovalbumin. The dot-plots show the intracellular IFNγ and CD40L staining of CD3^{+}CD8^{+}CD4^{-} or CD3^{+}CD8^{-}CD4^{+} gated splenocytes.

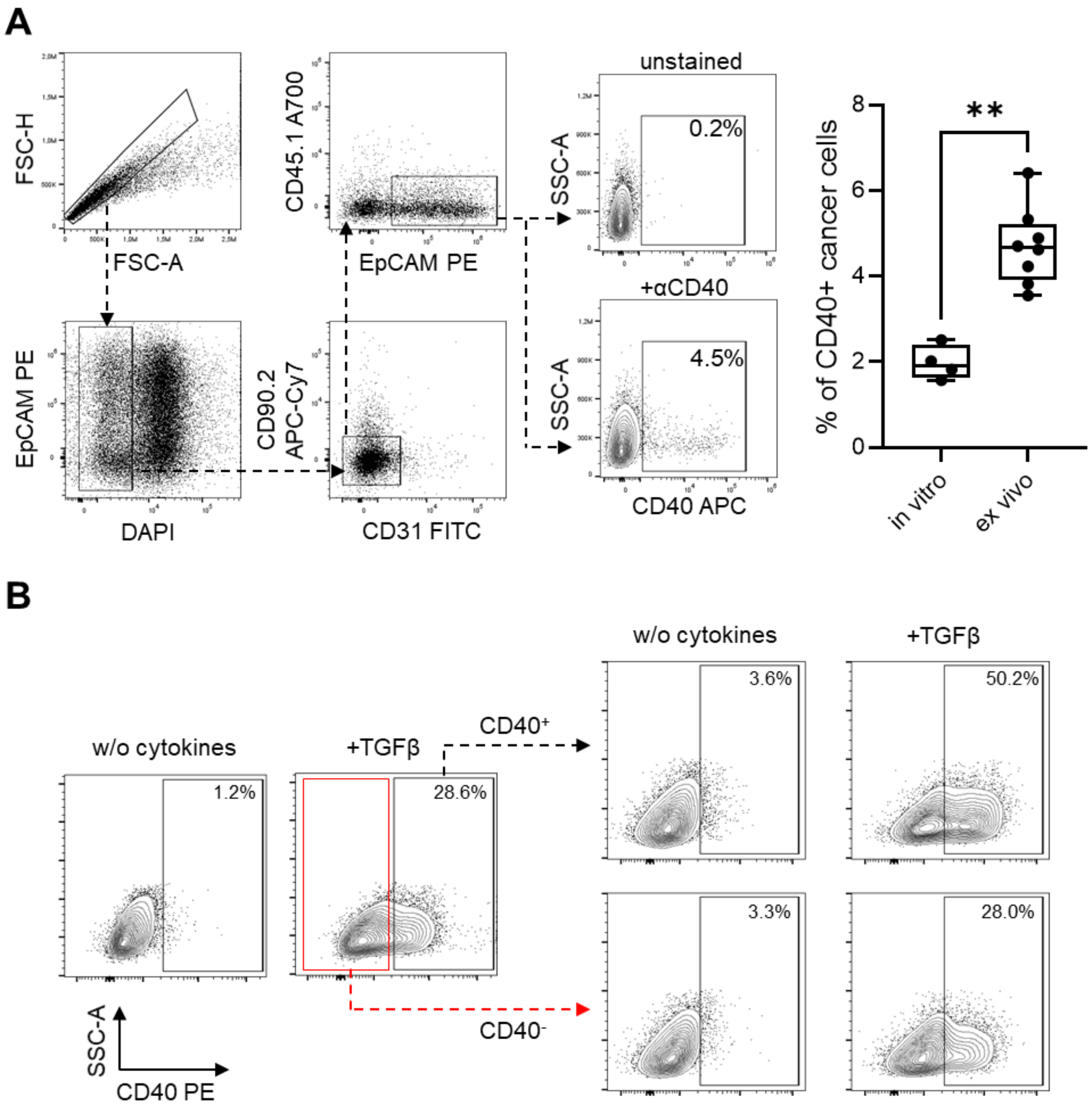

Supplementary Figure 6. Assessment of CD40 expression on 9.27-derived tumors and CD40 expression dynamics. A) CD40 expression on 9.27 cancer cells obtained from tumors grown for 16 days after s.c. injection into RAG1 KO mice was analyzed using flow cytometry. Shown is the gating strategy to assess CD40 expression on cancer cells (left) and a summary of the CD40 levels measured ex vivo on 9.27 cells of eight mice compared to CD40 expression levels on 9.27 cells from four in vitro cultures (right). B) $CD40^+$ and $CD40^-$ 9.27 cells were sorted after 24h TGFβ treatment. After 10 days of culturing without any cytokine supplement, both sorted fractions were treated again with TGFβ for 24h and CD40 expression was measured by flow cytometry. Statistical analysis: (**A**) Mann-Whitney U test: $p < 0.01$ = **.

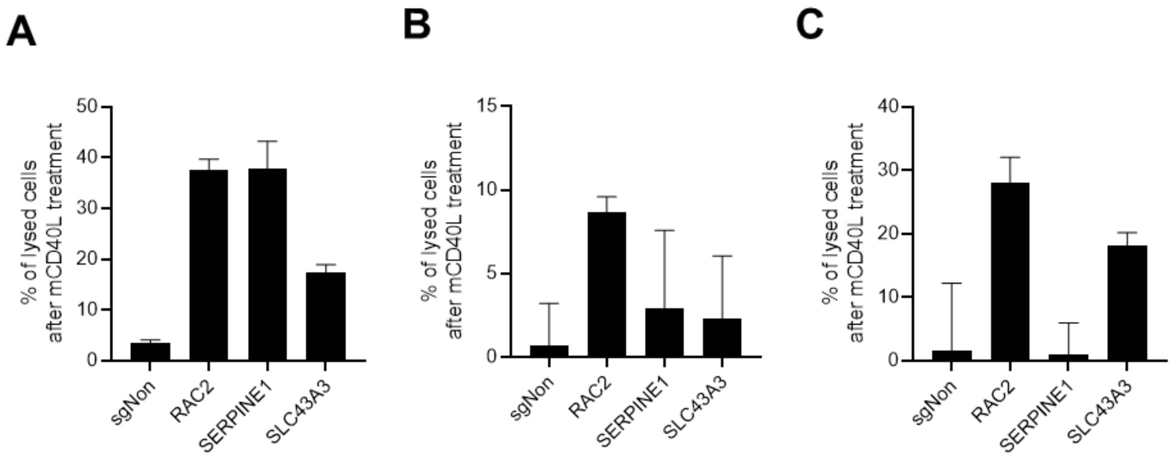

Supplementary Figure 7. CRISPR/Cas9 editing of RAC2, SERPINE1 and SLC43A3 sensitizes to CD40-mediated cell death. sgNon-targeted or CRISPR/Cas9 knockout 786O (**A**), BFTC909 (**B**) or KMRC1 (**C**) were treated with multimeric CD40L for 48h and specific lysis was determined by LDH release. Bars represent the mean specific lysis of triplicates of one out of two experiments.

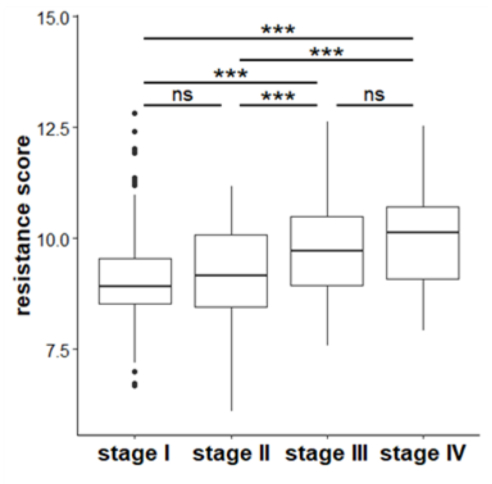

Supplementary Figure 8. The resistant score for CD40-mediated cell death increase during progressive tumor disease. The diagram displays the resistant scores of 532 patients based on the RNA sequencing data of the TCGA-KIRC dataset in relation to the RCC disease stages. Statistical analysis: ANOVA with Tukey multiple comparisons of means post hoc test was used (not significant = ns, $p < 0.001$ = ***).

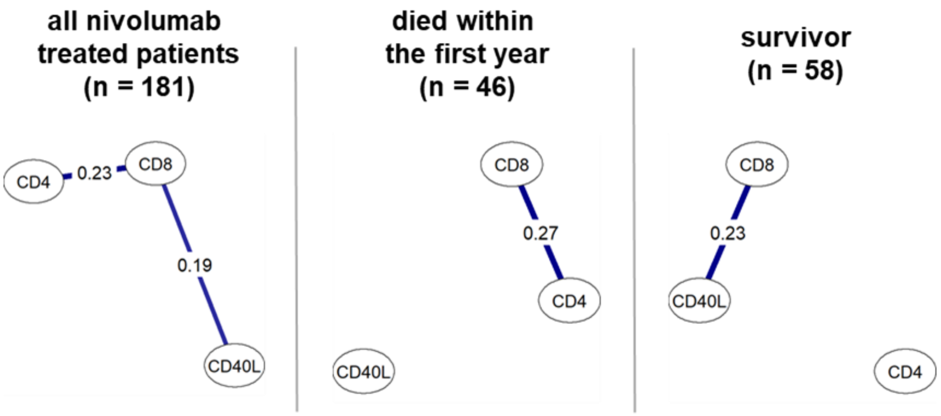

Supplementary Figure 9. Partial correlation networks between CD40L, CD8, and CD4 of RCC patients with stage IV tumors. Partial correlation networks of different patient groups treated with nivolumab are displayed and the numbers are the partial correlation coefficients. The data were obtained from the checkmate studies-009/ -010/ -025 [34].
